# Advancement from Small Peptide Pharmaceuticals to Orally Active Piperazine-2,5-dion-Based Cyclopeptides

**DOI:** 10.3390/ijms241713534

**Published:** 2023-08-31

**Authors:** Vladislav Deigin, Natalia Linkova, Olga Volpina

**Affiliations:** 1 The Laboratory of Synthetic Vaccines of Shemyakin-Ovchinnikov Institute of Bioorganic Chemistry, Russian Academy of Sciences, Miklukho-Maklaya St., 16/10, Moscow 117997, Russia; volpina@ibch.ru; 2 The Research Laboratory of the Development of Drug Delivery Systems, St. Petersburg Research Institute of Phthisiopulmonology, Ligovskii Prospect, 2-4, St. Petersburg 191036, Russia; miayy@yandex.ru

**Keywords:** immunotropic peptides, reciprocal activity peptide enantiomers, cyclic peptidomimetics, drug development

## Abstract

The oral delivery of peptide pharmaceuticals has long been a fundamental challenge in drug development. A new chemical platform was designed based on branched piperazine-2,5-diones for creating orally available biologically active peptidomimetics. The platform includes a bio-carrier with “built-in” functionally active peptide fragments or bioactive molecules that are covalently attached via linkers. The developed platform allows for a small peptide to be taken with a particular biological activity and to be transformed into an orally stable compound displaying the same activity. Based on this approach, various peptidomimetics exhibiting hemostimulating, hemosuppressing, and adjuvant activity were prepared. In addition, new examples of a rare phenomenon when enantiomeric molecules demonstrate reciprocal biological activity are presented. Finally, the review summarizes the evolutionary approach of the short peptide pharmaceutical development from the immunocompetent organ separation to orally active cyclopeptides and peptidomimetics.

## 1. Introduction

Peptides regulate multiple biochemical reactions in living organisms and are potential prototypes for numerous drug preparations [1]. The versatility of peptide functions in humans has made it possible, based on endogenous molecules and their modified analogs, to develop peptide pharmaceuticals for various indications, from oncology to dentistry [2]. In addition, natural peptides regulate emotional states, sexual behavior, sleep, wakefulness, and immune response. Modern technologies are used to isolate, synthesize, and study human, animal, and plant peptides as therapeutic agent candidates [3].

This review is devoted to the previously undiscovered properties of short peptides (mainly di-tetrapeptides) and pharmaceutical preparations based on them. Although the literature contains many comprehensive studies on the properties of low-molecular-weight peptides, including those based on 2,5-DKP [3,4,5,6], there are only eight pharmaceuticals based on various cyclopeptides that are approved for medical use [7], and not a single drug containing a 2,5-DKP derivative. 

This review describes short peptides’ discovery, development, and biological activities and their cyclic derivatives based on 2,5-DKP.

Few scientific publications, like [8], have described the activities of approved low-molecular-weight peptide drugs. 

In the reciprocal actions of the enantiomeric pairs of linear and cyclic peptides, which acted in strictly opposite directions both systemically and orally, we found no examples of such behavior in the available literature. The publications list on this topic is practically unavailable, and the phenomenon’s roots require further study.

Therefore, this review presents a topic that is not covered in the scientific literature—the original development steps from peptide extracts to peptide drugs Glu-Trp (Thymogen), Thymodepressin (D-Glu(D-Trp), and Stemokin (Ile-Glu-Trp) to orally active peptidomimetics based on the natural Glu-Trp peptide. In addition, a new approach to obtain orally active peptidomimetics based on a novel platform for synthesizing cyclic branched 2.5 DKP is discussed.

One of the essential biopharmaceutical areas is the multifunctional immuno- and hemoregulatory network, originating the permanent cyclic renewal process—from pluripotent stem cells to differentiated “matured” variety functional cells [9,10]. Since the 1960s, in the “pre-omics era,” the central organs of the animal immune system (bone marrow, thymus, and spleen) have attracted researchers’ attention as a source of biologically active compounds affecting the immune system. As far back as the middle of the last century, researchers suggested that some peptides may work as the immune system’s “hormones”. They discovered that the bone marrow, thymus, and spleen are the primary producers of such immune “hormones” [11]. In addition, some peptides participate in the maturation and differentiation of hematopoietic and immunocompetent cells [12]. 

The systematic biological activity studies of thymic peptides began in 1968 when A. Goldstein discovered that the thermostable thymus fraction prepared by the acetone extract from the crude thymus enhanced the suppressed immune response in mice. The first generation of immunomodulating preparations consists of extracts from the thymus and spleen was developed. One of the first preparations was “thymosin fraction 5”, a precursor of Thymosin alpha 1 (Zadaxen); the individual peptide was later isolated from the thymus extract [13]. In the following decades, several active peptides such as thymopoietin, splenopoietin, and thymulin isolated from thymic and spleen extracts were used in clinical practice as immunocorrecting agents [14]. 

## 2. Thymus-Derived Short Immunotropic Peptide Pharmaceuticals

### 2.1. First Generation: Thymogen 

Since the 1980s, researchers have also worked on isolating individual peptides from the calf thymus homogenate and determining their chemical structures and biological activities.

The isolation of low-molecular-weight peptides, especially from mammals’ organs and tissues, is challenging since the body contains multiple enzymes, including the specific dipeptidase. For example, as a rare case, the renal and intestinal brush borders enzyme *dipeptidase W*, which hydrolyzes X-Trp (X-W) dipeptides to amino acids, is not located in the thymus [15]. 

Using the preparative HPLC, a pool of ten Trp-containing dipeptides (Glu-Trp, Ile-Trp, Asp-Trp, Asn-Trp, Gln-Trp, Ser-Trp, Thr-Trp, Ala-Trp, Lys-Trp, and Gly-Trp) was isolated from the low-molecular-weight fraction of the pharmaceutical preparation of Thymalin (manufactured from the calf thymus extract). The test results show that the Glu-Trp dipeptide had the highest activity, and the peptides Ile-Trp, Asp-Trp, and Gln-Trp were moderately active. In contrast, other separated dipeptides were inactive in the screening test [16]. 

Since the natural peptides were isolated in milligram quantities, all the isolated peptides were synthesized and used for the study, with synthetic peptides identical to the natural ones [12,16,17,18]. The X-Trp-containing dipeptides were screened in vitro, exploring the active E-rosette test (Ea-RFU) [15]. The peptides isolated from the thymus and various synthetic isomers and analogs were used for the structure-functional (SAR) studies [17,18,19,20,21]. It is known that thymocyte treatment via trypsin results in the hydrolysis of most of the surface erythrocyte E-receptors of a ship or rabbit. As a result, the percentage of T-lymphocytes detected in the Ea—RFU test decreases. Adding the thymus peptide fractions or individual peptides (thymosin alpha 1) to the thymocyte culture promotes the regeneration and rapid recovery of E-receptors on trypsin-treated T-lymphocytes. The influence of peptides on the regeneration process of thymocyte E-receptors treated with trypsin was determined. Only Glu-Trp (Thymogen) was used as a leading compound for further experimental and clinical studies. The specific and general pharmacological activities of Thymogen were assessed in various experimental models [21,22,23,24,25].

### 2.2. The Regulation of Immunodeficiency Conditions by Thymogen

It is known that irradiation causes total damage to all vital cellular and tissue systems of the organism, which leads to a sharp decrease in both specific and non-specific defenses of the body. Therefore, Thymogen studies were conducted on animals exposed to ionizing radiation. Irradiation has a particularly destructive effect on lymphoid tissue, which is associated with its high radiosensitivity [26,27].

It was established that the exposure to ionizing radiation in all study periods decreased the numbers of karyocytes and T- and B-lymphocytes in the lymphoid organs of irradiated animals. To a greater extent, the violation of the cellular composition was noted in the thymus and lymph nodes. Along with this, there was an inhibition of the functional activity of lymphocytes and neutrophils in the blood of irradiated guinea pigs, as evidenced by a 1.5- to 2-fold increase in the percentage of leukocyte migration and a 20–35% decrease in the number of cationic proteins in neutrophils [27]. 

The treatment by Thymogen prevented a decrease in the numbers of karyocytes and T- and B-lymphocytes in the lymphoid organs of irradiated animals. The drug had the most pronounced effect on the thymus and, to a lesser extent, on the lymph nodes and spleen. In addition, Thymogen prevented an increase in the percentage of leukocyte migration on day five after irradiation and normalized this indicator by day 20 after radiation exposure compared to the control. 

Thus, the results show that Thymogen stimulated the restoration of the numbers of karyocytes and T- and B-lymphocytes in the lymphoid organs of irradiated animals and normalized the functional activities of blood lymphocytes and neutrophils [26,27].

The Thymogen’s detailed molecular mechanism is still the subject of active research. However, suppose the thymopoietin, splenin, and thymulin protein receptors of cell membranes are considered a target [11]; in the case of Thymogen, it targets the promoter regions of the DNA double helix in lymphocyte cells. It is assumed that such binding transforms the “silent” heterochromatin into active euchromatin, which increases the availability of respective genes for transcription [19]. Subsequently, dipeptide GluTrp displayed a broad range of immunopotentiation effects and, after preclinical and clinical studies, was registered in 1990 as a drug Thymogen in the USSR (now in the Russian Federation) (Russian Ministry of Health Registration Certificate № P N002408/01 from 10 June 2009). 

### 2.3. Second Generation: Stemokin 

In the SAR studies of synthetic Glu-Trp family peptides, the tripeptide Ile-Glu-Trp showed significant immuno- and hemostimulating effects in mice model experiments. This peptide stimulated the exogenous bone marrow colony restoration after irradiation in 1Gy and 4Gy doses, while Thymogen reduced the radiation effects only at 1 Gy [27,28]. Given the close interaction of the hematopoiesis and immune system [29] consisting of a common ancestral cell and for the regulation of specific processes of hematopoiesis by immunocompetent cells, it was of particular interest to study the possibility of using short peptides to reduce the damaging effect of ionizing radiation on hemopoiesis, particularly on the undeveloped CFU cell population [19]. The impact of Ile-Glu-Trp was compared with the Granocyte (colony-stimulating factor G-CSF) on hematopoietic cells after a higher irradiation dose of 7 Gy. On the 14th day after irradiation, both the peripheral and initial departments of hemopoiesis in the treated groups were recovered. Under the influence of Ile-Glu-Trp, by day 14, the volume of the CFU pool increased by 2.5 times. By day 21 after irradiation, none of the experimental groups showed a significant difference in the cellular composition of peripheral blood from the intact control. Currently, in the bone marrow, the volume of the CFU pool in mice treated with Ile-Glu-Trp is completely restored, significantly outperforming the group treated with G-CSF, in which the amount of CFU was two times less than in the intact control. Thus, the restoration of hematopoiesis in irradiated mice (7 Gy) under the influence of Ile-Glu-Trp and G-CSF is observed from the 14th day, which is much earlier than in the control group. In addition, Ile-Glu-Trp restored the total amount of bone marrow cell faster than the G-CSF group. Moreover, the cellularity volume in the Ile-Glu-Trp-treated group was completely recovered two times more intensively than in the group treated with G-CSF [30].

Compared to synthetic di-hexapeptides, the tripeptide Ile-Glu-Trp showed pronounced immuno- and hemostimulating effects in the mice model of the exogenous colony restoration after irradiated bone marrow [31]. Based on the preclinical and clinical studies, the peptide Ile-Glu-Trp was registered in Russia as a drug named Stemokin (Russian Ministry of Health Registration Certificate № LCP-003014/09 from 16 April 2009). 

### 2.4. Third Generation: Thymodepressin

In the continuation of the SAR studies on the biological activity of various isomers of the Glu-Trp dipeptide, the original data on the influence of peptides on the regeneration process of thymocyte E-receptors treated with trypsin were determined. Several peptides exhibited statistically significant activity, accelerating the restoration of thymocyte ability in Ea—RFU formation. Some peptides were inactive in this test system, and two peptides—D-Glu-D-Trp and D-Glu-(D-Trp)—unexpectedly showed the opposite activity. The addition of these peptides led to a further decrease by almost twofold in the number of Ea—RFU of T-lymphocytes treated with trypsin. It was assumed that the action of these two peptides results in blockage, the restoration of the number of destroyed E-receptors, and, consequently, a decrease in the number of rosette-forming cells [16].

The reasons for the unique activity reversal found for D-Glu-D-Trp and D-Glu-(D-Trp) peptides required further investigation, and a set of experiments in a wide range of conditions with various doses of Thymodepressin in combination with and without cytostatic and allogeneic bone marrow were performed [31]. A systemic study of Thymodepressin in cell cultures and animal models concluded that bone marrow hematopoietic progenitors are the primary target of its action [32].

#### 2.4.1. Thymodepressin Influences the Development of Hemopoietic Precursor Cells Development 

In further experiments, the hemosuppressive activity of Thymodepressin was studied on the proliferation of the hemopoietic precursors in vitro. The study materials were hematopoietic precursor cells isolated from donor bone marrow and the umbilical cord blood of premature infants at 35–36 weeks’ gestation using the mitochondria toxicity assay (MTT assay) in methylcellulose. The effect of Thymodepressin on the cultivation of hematopoietic progenitors of human cells was studied in parallel with a control for each study without adding Thymodepressin. The test result indicates that Thymodepressin doses from 10 µg/mL to 1 µg/mL suppress the cloning efficiency of all types of hematopoietic progenitors [33].

#### 2.4.2. Thymodepressin Effects on Hemopoiesis and Immunity

Stem cells were used in the in vivo study on the spleen colony formation at the early steps of hemopoietic precursor proliferation (CFU-S). These proliferating cells form colonies on the spleen; only pluripotent cells mature on the 12th day of growth. More committed pooled cells gave rise to colonies that matured on the eighth growing day (CFU-S-8). In this model, the relative amount (per 10^5^ cells) of CFU-S-8 in the bone marrow of donor mice was determined [34]. Thymodepressin, both in vitro and in vivo, affects the initial stages of hemopoiesis, reducing the number of committed (CFU-C-8 and CFU-GM) cells and the percentage of cells in the S-phase of the cell cycle. As a result, the administration of the peptide leads to a dose-dependent transient decrease in the number of leucocytes in the blood of the experimental animal. The primary method used to prove this mechanism of action was applied compared to the sensitivity of normal mouse bone marrow cells [33]. In addition, a direct measurement of the radiation allows one to correctly measure the percentage of dividing cells (in the S phase of the cell cycle) [33].

The experimental and clinical data show that Thymodepressin has immunosuppressive activity in the models treating autoimmune and allergic processes caused by lymphocyte-mediated hyperimmune reactions:

- The peptide D-Glu(D-Trp)-OH inhibits the migration of CD34+ cells from the bone marrow into peripheral blood in normal and tumor-bearing animals [31]. Injecting this peptide into donor mice two days before irradiation or the exposure to the cytostatic cytosine arabinoside (cytosar) D-Glu(D-Trp)-OH protected the population of hematopoietic progenitor cells and promotes more intensive restoration than the control group [32].
- A direct suppression of the autoimmune reaction in the mice models by the peptide D-Glu(D-Trp)-OH more effectively prevented and treated the developed autoimmune reactions when directly compared with Cyclosporin [32].
- Therefore, after preclinical and clinical studies, the peptide D-Glu(D-Trp-OH) was registered in the Russian Federation as a drug named Thymodepressin^®^ (Russian Ministry of Health Registration Certificate № LCP-001836/08 17 March 2008). The immuno-inhibiting properties of this unnatural iso-peptide were in demand in medicine. Currently, Thymodepressin is actively used in clinical practice for the treatment of autoimmune and allergic processes caused by lymphocyte-mediated hyperimmune reactions; these include psoriasis, atopic dermatitis, lichen planus, autoimmune cytopenia, and other syndromes [25,32].

## 3. Toward a Fourth Generation of Glu-Trp Family Peptide Pharmaceuticals

### 3.1. Discovery of the Reciprocal Activities of Glu-Trp Family Peptides

In further SAR studies of the Glu–Trp peptide family, the rosette-forming units’ (RFUs) recovery screening model revealed the different influences of the chemical and optical configurations of each dipeptide constituent amino acid on the direction and intensity of the biological activity of the single molecule. Furthermore, the configuration reversal of both chiral centers led to an unusual result: both D-D isomers, D-Glu-D-Trp (α-bond) and (D-Glu)-D-Trp (γ-bond), did not only show immunostimulating activity, but also proved to be effective inhibitors of regeneration under the same conditions in which the L-L-isomers showed stimulating activity [10,21].

As a result, it was discovered that L-Glu–L-Trp-OH, L-Glu–(L-Trp)-OH, and their structural and optical isomers D-Glu-D-Trp-OH and D-Glu(D-Trp)-OH exhibit reciprocal effects on thymus-derived cells (thymocytes) in vitro; the L–L-peptides had immunostimulating properties, while the D–D-peptides had immunosuppressive properties. Furthermore, in the in vivo experiments in mice, the L-Glu–L-Trp and its structural and optical D-isomer D-Glu(D-Trp)-OH had similar reciprocal effects to the in vitro experiments on immunocompetent cells and hemopoietic progenitor cells; the L–L-peptide possesses hemostimulating properties, while the D–D-peptide possesses hemosuppressive properties [10]. D-Glu-D-Trp-OH and D-Glu(D-Trp)-OH peptides’ surprising activities were further investigated. Therefore, a comprehensive range of experiments was carried out in various conditions and doses of Thymodepressin in combination with and without cytostatic and allogeneic bone marrow transplantation [10,21,35]. 

### 3.2. Development of 2,5-DKP-Based Peptidomimetics

The unique influence of optical isomers on the functional characteristics of the Glu-Trp family’s immunoregulating peptides has stimulated research to expand this finding to other small peptide molecules. One of the significant disadvantages of peptides is that an intravenous (IV) administration limits their use in medical practice due to low stability under non-invasive administration. Various critical issues associated with therapeutic peptide delivery have drawn attention to developing new formulations for alternative routes of administration, such as oral, nasal, buccal, pulmonary, transdermal, rectal, and ocular [36].

The approaches that increase peptide stability continuously improve, leading to new structural modifications [37,38,39,40,41]. Among the standard ways of increasing the enzymatic stability of peptide molecules is to introduce elements with a cyclic structure that is resistant to proteolytic degradation [37,38]. Many laboratories worldwide are developing peptide modification strategies to increase the stability and affinity to receptors or active enzyme centers [42,43,44,45]. The minimal cyclic structures of peptide compounds are cyclodipeptides (2,5-diketopiperazines (DKPs), which are obtained by condensing two alpha-amino acids [46].

2,5-DKP is resistant to proteolysis and is attractive for structural and functional studies searching for new potential drugs. The conformationally limited chiral centroid structure of 2,5-DKP enables alterations at all six positions and enables stereochemical isomerization at all four optical centers. In addition, 2,5-DKP has a rigid framework that can mimic the preferred conformation by limiting the mobility of amino acids embedded in its structure. Furthermore, the large set of substituents makes it possible to significantly vary the molecule’s physicochemical characteristics, such as its structure, size, shape, lipophilicity, dipole moment, electrostatic charge, and functional groups [46,47,48,49]. An approach implying the use of 2,5-DKPs, both in short peptide analogs and in the form of “inserts” at different positions of the peptide chain, has become widespread to increase their hydrolytic stability and the possibility of oral administration. Several cyclopeptides are approved for clinical use as peroral medications [47,48,49].

**Figure 1:**
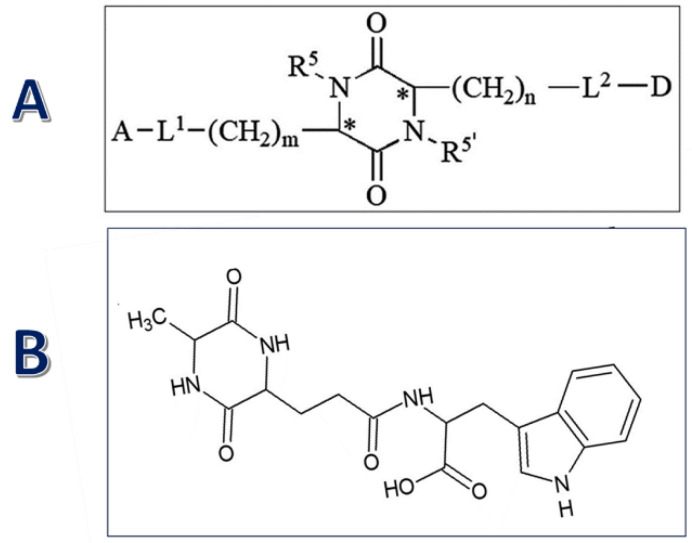
The general formula of peptidomimetics platform (A) and peptidomimetic AWE18. (**A**) For synthesizing libraries, A and D represent biologically active pharmacophores or fragments of peptide compounds; L^1^ and L^2^ are biodegradable linkers; m and n are the numbers of CH_2_ groups (ranging from 0 to 4); and R^5I^ and R^5^ represent possible derivatives of the pharmacophore attached to the nitrogen atoms. * Indicates that an S or R optical orientation is possible at the carbon atoms at positions 3 and 6 [50]. (**B**) Formula for active immuno- and hematopoiesis, stimulating cyclopeptide AWE18.

Moving to the next generation of reciprocally active peptides, we developed an original platform for modifying Glu-Trp-based peptides by synthesizing substituted 2,5-DKP peptidomimetics (Figure 1). These derivatives of the 2,5-DKP consist of a trifunctional amino acid containing various functional groups, which can be used to identify target positions for interaction or as linkers for attaching multiple pharmacophores [50]. 

In the next advancement for our peptide pharmaceutical development, this platform was applied to the design and synthesis of orally active immune- and hemoregulating Thymogen-Thymodepressin analogs [39,40,41]. The unmet need for safe suppressor preparations led several researchers to develop down-regulating medications that suppressed the immune- and hemopoietic cell cycle processes. The combined hemo- and immunoregulating data on novel Thymogen-Thymodepressin analogs have created the background for extending our platform technology of exploring DKP peptidomimetics in broad areas of unmet medical needs [41].

## 4. Development of the Peptidomimetic [AlaGlu(Trp)]

A library of cyclic peptidomimetics was synthesized for SAR. Several peptides exhibited immunosuppressive activity [39], and their enantiomeric analogs exhibited immunostimulatory activity [40,41]. Studies of cyclic peptidomimetics and their linear precursors in various in vitro and in vivo models have identified the potential drug candidate cyclopeptide [AlaGlu(Trp)] (code AWE18) for further experimental studies. This peptidomimetic showed activity parenterally and orally in several experimental models [41]. A comparison of the actions of pharmaceutical preparations of Stemokin and Thymogen with AWE18 showed the advantage of this peptidomimetic, considering that Stemokin and Thymogen are active only in systemic and intranasal applications, representing the first generations of peptide stem cell stimulants. 

Its creation suggests the emergence of a new perspective—“endogenous” ways of applying stem cell therapy. The test results reveal that AWE18 possesses a similar spectrum of biological activity with Stemokin and Thymogen and stimulates the proliferation of intact stem cells in systemic and oral application [40,41]. 

Thymogen, when administered parenterally, stimulates the differentiation and proliferation of T-lymphocytes and promotes the activity of neutrophils and monocytes, providing a regulatory effect on cellular and humoral immunity [10,22,26]. It was also shown that Thymogen stimulates the proliferation of damaged bone marrow cells [17]. Stemokin exhibits an increased affinity to bone marrow cells and is proven to be a hemostimulating immune and adjuvant agent [27,30]. 

**Figure 2:**
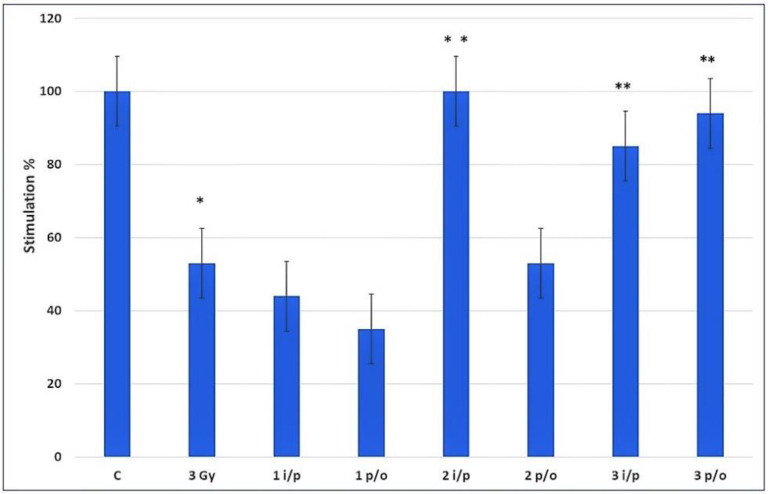
Comparative hemostimulated activities of Thymogen (1), Stemokin (2), and AEW18(3) [27]. * *p* < 0.05 vs. control mice; ** *p* < 0.05 vs. irradiated mice; i/p—intraperitoneal; p/o—per/os administration. The ratio of the cell colony number on the recipient spleen versus the control (in %) is shown.

Further experiments based on the measurement of the colony-forming ability of undifferentiated (intact) and damaged bone marrow stem cells in the spleen of sublethally irradiated recipients (exogenous spleen support) were performed to compare the influences of AWE18, Thymogen, and Stemokin on the initial studies of hemopoiesis on intact bone marrow and on bone marrow damaged by 1 Gy irradiation. In this test, the administration of AWE18 systemically (i/p, intraperitoneally) and (p/o, orally) statistically significantly increased the number of colonies by more than 43–52% (Figure 2) [27,40].

Using the different models, the tested peptides were studied on damaged bone marrow via 3 Gy irradiation. Bone marrow suspension from femoral marrow was irradiated ex vivo; irradiated and control suspensions were administered intravenously to lethally irradiated recipients. As a result, AWE18 restored the population of CFU-S-8 affected by irradiation close to the level of intact control [40].

The treatment of bone marrow cells taken from the intact animals via ex vivo mild irradiation (3Gy) leads to the depletion of colonies. Therefore, the irradiated or control suspension was injected in the tail vein into lethally irradiated (8 Gy) recipient mice. The studied peptides were administered to recipient animals intraperitoneally (i/p) or orally (p/o) 1 h after an injection of the irradiated bone marrow. The results demonstrate that the linear peptides, Thymogen and Stemokin, showed a high stimulation at systemic application, and did not show activity in the post-radiation cell restoration in oral administration. In contrast, AWE18 offers increased activity in both ways of application, which is commensurate with the bioactivities mentioned above for Thymogen or Stemokin.

After the AWE18 toxicology study in two experimental mice models, the acute (single administration) and chronic (three-month daily parenteral administration) test results show the following conclusions: the preparation is not toxic to the animals after a single parenteral injection (i.p.) to the maximum dose tested—10,000 µg/kg in mice. 

The recommended potential human therapeutic dose of AWE18 is about 10–100 µg/kg, while the amount tested in mice showed no toxic effect by at least 10–1000 times. The results of the toxicological tests suggest that the extrapolation of AWE18 can be safe after a single and course administration at doses of up to 100 µg/kg. 

## 5. Discussion 

This review describes approaches to the creation of effective pharmaceutical preparations based on the natural dipeptide Glu-Trp and its linear and cyclic analogs, consisting of modifications of the molecular structure via conversion into derivatives of substituted (“branched”) 2,5 diketopiperazines with different optical orientations of constituent amino acid derivatives.

Peptides represent a unique therapeutic niche and are prominent in emerging biopharmaceuticals. However, obtaining individual peptide preparations has been challenging since the isolation and synthesis of the first peptides. By understanding the prospect of using peptides as tools for solving problems associated with improving the specifics of drug action and searching for new compounds and their analogs, peptide chemists took the risk of turning this challenge into an opportunity in the early 1980s. 

Over time, chemical synthesis technologies have been significantly improved, and new methods for modifying molecules have appeared, including using non-natural amino acids, introducing pseudopeptide bonds, and other modifications [10,48,49,51,52]. Natural peptides have a low cell membrane permeability, limited stability in vivo, and low oral bioavailability, so their preferred administration route into the body is via subcutaneous, intramuscular, or intravenous injections [53].

Unmodified peptides have a short half-life, and their activity is usually limited to extracellular targets. The low oral bioavailability of unmodified peptides is caused by proteolysis in the blood, kidneys, or liver and by rapid renal clearance during the gastrointestinal tracts and liver’s initial passages [54,55,56,57]. 

The approaches that increase peptide stabilities continuously improve, leading to new structural modifications [58]. 

Despite the difficulty of developing orally stable peptides and peptidomimetics, many laboratories are intensively developing such pharmaceuticals [58,59]. One apparent solution for stabilizing the hydrolytic lability of natural peptides is incorporating modified analogs of natural peptides, which were previously registered as parenteral drugs [53,54,55]. Analog modifications are based on substitutions in various parts of the original molecule to stabilize and sometimes change its structure, spectrum, and even direction of action [44]. An essential requirement for improving peptide structures is minimizing the possible toxicity of the obtained analogs. 

The chemical “stapling” of amino acid side groups into a peptide chain can be achieved by “stapling” side amino acid residues in a peptide chain with hydrocarbon “inserts” or by forming lactam bridges to stabilize the helicity and increase the stability and intracellular permeability of peptides. The so-called “stapled-peptides” method is gaining popularity [38]. 

A new concept of creating full-length enantiomeric D-peptides that substitute all corresponding L-amino acids became widespread. Such peptides (D-peptides) increase the half-life of the target product and significantly improve its stability [58,60,61].

The approaches used to increase peptide stabilities are continuously being improved, leading to new kinds of structural modifications [54,55,56,57,58,59,60,61]. One apparent solution for stabilizing the hydrolytic lability of drugs containing natural peptides is incorporating modified analogs of natural peptides that were previously registered as parenteral drugs. Analog modifications are based on substitutions in various parts of the original molecule to stabilize and sometimes change its structure, spectrum, and even direction of action [62], [63].

An essential requirement for improving peptide structures is minimizing the possible toxicity of the obtained analogs. Many laboratories worldwide [60,61] are developing peptide modification strategies to increase receptor stability and affinity. 

Another modern approach to increase the peptide stability and create a more durable compound is to explore the different cyclization techniques [64,65,66].

**Figure 3:**
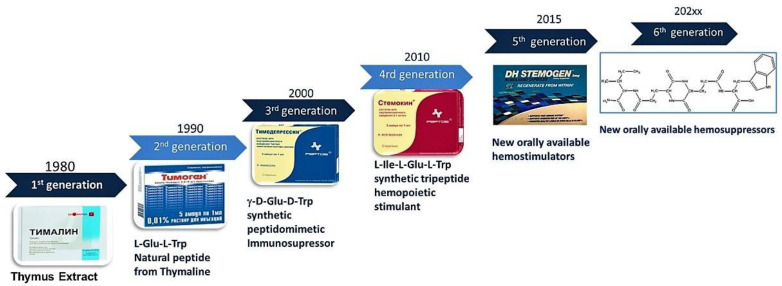
Evolution of immunoactive peptide pharmaceuticals.

Over many years, peptide researchers have developed and introduced several generations of peptide drugs that are active in parenteral and intranasal administration as a new perspective on orally available peptidomimetics that could appear in the pharmaceutical market [Figure 3]. 

The modern trends for developing the next generation of peptide pharmaceuticals demonstrate a growing demand for non-invasive, preferably oral, drug forms [67,68].

The first Glu-Trp dipeptide, isolated from calf thymus extract, possessed immunostimulating properties, and a drug based on it has been sold in Russia for more than 30 years [22].

As a result of SAR, original analogs were obtained, and based on the Ile-Glu-Trp tripeptide, the hemostimulator Stemokin was developed, acting at the level of hematopoietic stem cells [27,28]. The study of isomers and analogs of the dipeptide Glu-Trp showed that the enantiomeric peptides LL-Glu-Trp and DD-Glu-Trp exhibit a reciprocal effect on immuno- and hemoregulation at the cellular level; LL-Glu-Trp peptides have a stimulating effect, and DD-Glu-Trp peptides exhibit suppressor properties [10]. 

This discovery enabled the creation of the original immunosuppressor Thymodepressin based on the dipeptide D-Glu(D-Trp) [10,32]. 

All three drugs have been patented and sold in Russia for many years.

We did not find similar works in open access journals. In the continuation of SAR, we took a novel approach to creating potentially orally active derivatives based on modified 2,5-DKP products [67].

Studies on the stability of such compounds in animals with oral and systemic administration have been carried out. An experimental comparison of the activity of linear and cyclic analogs of LL-DD-derivatives showed that the reciprocal effect is retained for some pairs of enantiomers [39,40,41].

As described above, cyclic 2,5-DKP molecules are convenient tools for various modifications and the in silico structure modeling for the targeted library design of 2,5-DKP derivatives [37,68].

An approach using 2,5-DKP derivatives [66] both in the form of short peptide analogs and in the form of “insertions” into different positions of the chain became widespread, with a variation in the location of 2,5-DKP at the *N*- and *C*-terminus of the molecule or inside the peptide chain to increase the hydrolytic stability and the possibility of oral administration. Examples of such modification of a diketopiperazine ring by attaching peptide fragments are described in [57,63,66,69,70].

Several cyclopeptide drugs have been approved worldwide [7]. Recently, a new synthetic cyclopeptide, plitidepsin (Aplidin^®^), was approved to treat multiple myeloma [67], and another it application was submitted to clinical trials as a promising antiviral agent against COVID-19 [68]. Another cyclopeptide. Plecanatide (Trulance) was approved for the treatment of Chronic Idiopathic Constipation and Irritable Bowel Syndrome with Constipation [71]. 

In the SAR process, a novel chemical platform for our Glu-Trp family peptides based on branched piperazine-2,5-dione derivatives (2,5-di-diketopiperazine) was developed to prepare protease-resistant non-invasive biologically active peptidomimetics [39,40,41].

The new generation of peptidomimetics has planned to solve the problem of the fast enzymatic degradation of Glu-Trp-based peptide drugs upon oral administration, exploring the original approach to convert dipeptides into 2,5-DKP using trifunctional amino acids as a universal scaffold [39,40,41]. 

Knowing the effect on the proliferation rate and inducing the differentiation of blood cells through our linear peptide Stemokin, the impact on initial hemopoiesis with Stemokin activity has been compared with cyclic peptides [20].

The results demonstrate cyclic peptides’ high activity in systemic and oral applications. Several cyclic analogs showed high hemosuppressive activities that are comparable to Thymodepressin in these tests. As a result, the branched DKP containing the D-Glu(D-Trp) fragment exhibited biological properties similar to linear structures upon parenteral and oral administration [40,41].

The experimental and clinical results of the activity of the peptides Thymogen, Stemokin, and Thymodepressin have created new opportunities for continuing and expanding the range of studies of a new generation of peptidomimetics [41]. 

In the exploration of the close interaction of the immune and neuroendocrine systems [72], a library of branched diketopiperazine derivatives was screened in neuroprotective and adjuvanticity experimental models. Thus, cyclic analogs, including AEW18, protected neuroblastoma cells from the damage of beta-amyloid (Aβ peptides) [41].

The evolutionary approach to studying short immunotropic peptides showed the perspective of a broader study of diverse types of biological activity besides the immunotropic peptides.

However, this approach was only applied to one type of peptide, the Glu-Trp family, and the discovery of the reciprocal action of enantiomeric dipeptides and their cyclic derivatives. The prospect of oral cyclopeptides and peptidomimetics is desirable for potential use in clinical practice. 

Typically, there are significant limitations, such as a low intracellular permeability and bioavailability [73]. Hydrogen bonds, lipophilicity, size, flexibility, and structure are the key factors affecting peptides’ passive permeability. Lipophilicity cyclic peptides require a degree of lipophilicity to cross peptides and the membrane—they can neither be too lipophobic nor too lipophilic. The backbone cyclic absence of free N- and C-termini reduces potential interactions between the peptide and solvent, which is desirable for permeability. A cyclic structure can also be more compact than a linear one, reducing its collision profile in the solution and allowing for it to diffuse faster through the membrane. Finally, cyclic peptides are typically more stable against chemical or enzymatic degradation than linear peptides. Therefore, more peptides are available at each step of the transport process, resulting in a more significant overall amount that is transported [74]. 

In this review, the cyclic structures are formed by dipeptides; their scaffold corresponds to the optimal parameters for permeability and absorption upon contact with the mucosa. Such modified minimal natural structures are optimal for preventing enzymatic degradation and for retaining their form after oral transport into the body. Our studies have shown the stability of 2,5 DKP upon hydrolysis by fragments of the organs of the gastrointestinal tract of rat derivatives [75]. 

To understand the broad applicability of this action for other short peptides with different types of biological activities, new studies and, possibly, other options for detecting a reciprocal effect on their biological properties will be required.

## 6. Conclusions and Prospects

The development of pharmaceutical preparations based on the Glu-Trp dipeptide is a practical approach to creating pharmaceuticals for treating pathological conditions of the immune and hematopoietic systems. The direct modification of the chemical structure and optical orientation of the components of amino acids allows for preparations with original immuno- and hematopoietic properties to be obtained. The data presented in this review show the possibility of using the described approaches to create new, previously undescribed pharmaceutical preparations.

The progress in the theoretical prediction of potential precise targets for natural and synthetic peptide pharmaceuticals makes it possible to predict and select targets for creating new peptide drugs. As a result, these new-generation molecules already have a perspective in several essential application areas in medical practice. Further harmonizing the regulatory field and industrial practice standards could help to expedite the delivery of a new generation of effective peptide-based pharmaceuticals.

These studies pave the way to introduce the next generation of peptide biopharmaceuticals into clinical practice by providing a new approach to expand the variety of peptide modifications via branched 2,5-DKP.

## Data Availability

No new data were created.
